# Mitochondrial reactive oxygen species generation in blood cells is associated with disease severity and exercise intolerance in heart failure patients

**DOI:** 10.1038/s41598-019-51298-3

**Published:** 2019-10-11

**Authors:** Ryosuke Shirakawa, Takashi Yokota, Takayuki Nakajima, Shingo Takada, Miwako Yamane, Takaaki Furihata, Satoshi Maekawa, Hideo Nambu, Takashi Katayama, Arata Fukushima, Akimichi Saito, Naoki Ishimori, Flemming Dela, Shintaro Kinugawa, Toshihisa Anzai

**Affiliations:** 1 Department of Cardiovascular Medicine, Faculty of Medicine and Graduate School of Medicine, Hokkaido University, Sapporo, Japan; 2 Xlab, Center for Healthy Aging, Department of Biomedical Sciences, University of Copenhagen, Copenhagen, Denmark; 3 Department of Geriatrics, Bispebjerg-Frederiksberg University Hospital, Copenhagen, Denmark

**Keywords:** Heart failure, Energy metabolism

## Abstract

Systemic oxidative stress plays a key role in the development of chronic heart failure (CHF). We tested the hypothesis that mitochondrial reactive oxygen species (ROS) generation in circulating peripheral blood mononuclear cells (PBMCs) contributes to CHF progression. A total of 31 patients who had a history of hospital admission due to worsening HF were enrolled and grouped as having either mild CHF defined as New York Heart Association (NYHA) functional class I-II or moderate-to-severe CHF defined as NYHA functional class III. ROS levels in PBMC mitochondria were significantly increased in CHF patients with NYHA functional class III compared to those with NYHA functional class I-II, accompanied by impaired mitochondrial respiratory capacity in PBMCs. ROS generation in PBMC mitochondria was positively correlated with urinary 8-hydroxydeoxyguanosine, a systemic oxidative stress marker, in CHF patients. Importantly, mitochondrial ROS generation in PBMCs was directly correlated with plasma levels of B-type natriuretic peptide, a biomarker for severity of HF, and inversely correlated with peak oxygen uptake, a parameter of exercise capacity, in CHF patients. The study showed that ROS generation in PBMC mitochondria was higher in patients with advanced CHF, and it was associated with disease severity and exercise intolerance in CHF patients.

## Introduction

Chronic heart failure (CHF) is a progressive clinical syndrome characterized by systemic illness such as anemia, renal failure, vascular endothelial dysfunction, and skeletal muscle dysfunction as well as cardiac dysfunction after initial onset of acute heart failure^1–4^. In particular, exercise intolerance is a cardinal symptom of CHF, and reflects a poor systemic physical condition leading to worse clinical outcomes^5^. Growing evidence suggests that systemic oxidative stress plays a key role in the development of CHF and can be used as an indirect and non-specific marker to predict disease severity and prognosis in these patients^6–8^. However, the underlying mechanism of enhanced systemic oxidative stress, including the origin of reactive oxygen species (ROS) in advanced CHF, remains fully unknown.

It has been shown that mitochondrial dysfunction is involved in CHF^9–11^. Mitochondria are a major source of ROS, and ROS-damaged mitochondria generate more ROS, in a self-reinforcing process known as “ROS-induced ROS generation”^11^. It was previously shown that CHF patients had a higher proportion of mitochondrial ROS-loaded white blood cells^12^. Among white blood cells, peripheral blood mononuclear cells (PBMCs), i.e., lymphocytes and monocytes, rely on mitochondrial oxidative metabolism, while neutrophils do not^13^. Previous studies have shown that mitochondrial dysfunction in PBMCs is related to the pathogenesis or development of a wide variety of chronic diseases, such as autism, depression, and amyotrophic lateral sclerosis^14–16^. In addition, a recent report demonstrated that mitochondrial respiratory capacity in PBMCs was depressed in patients with early-stage CHF (asymptomatic patients)^17^, but the role of PBMC mitochondrial dysfunction in the development of CHF remains unclear.

Here we focused on mitochondrial dysfunction in PBMCs, which have a higher demand for oxidative metabolism, and hypothesized that increased levels of mitochondrial ROS in circulating PBMCs might contribute to the development of CHF. The aims of this study were to compare the mitochondrial ROS generation in circulating PBMCs in patients with mild CHF defined as New York Heart Association (NYHA) functional class I-II or moderate-to-severe CHF defined as NYHA functional class III, and to investigate its association with disease severity and exercise intolerance in CHF patients.

## Results

### Baseline characteristics

Baseline data of the CHF population are presented according to NYHA functional class in Table 1. There was no significant difference in age, gender, body weight, body mass index, cardiovascular risk factors, primary cause of HF, or medications between the NYHA I-II and NYHA III groups. There was no significant difference in urinary 8-hydroxydeoxyguanosine (8-OHdG), a systemic oxidative stress marker, between these groups. The plasma level of B-type natriuretic peptide (BNP), an established biomarker of HF severity, was significantly higher in the NYHA III group than the NYHA I-II group. The left ventricular ejection fraction (LVEF), a parameter of left ventricular systolic function, was significantly lower in the NYHA III group. In addition, the NYHA III group had a reduced peak oxygen uptake (VO_2_) compared to the NYHA I-II group, suggesting exercise intolerance.

**Table 1:** Demographic and clinical characteristics of CHF patients with NYHA functional class I-II and NYHA functional class III.

	NYHA I-II (n = 15)	NYHA III (n = 16)	*P*-value
Age, years	63 ± 13	61 ± 14	0.637
Male	14 (93)	15 (94)	0.962
Body weight, kg	66.6 ± 11.1	66.9 ± 14.7	0.948
Body mass index, kg/m^2^	24.4 ± 3.5	24.1 ± 4.5	0.837
**Primary cause of heart failure**			
Ischemic	4 (27)	7 (44)	0.321
Non-ischemic	11 (73)	9 (56)	0.321
**Cardiovascular risk factors**			
Hypertension	5 (33)	1 (6)	0.057
Diabetes mellitus	4 (27)	7 (44)	0.321
Dyslipidemia	6 (40)	10 (63)	0.210
Urinary 8-OHdG, ng/mg Cr	9.3 ± 2.8	11.5 ± 6.1	0.198
Plasma BNP, pg/dL	101.8 ± 105.0	286.2 ± 240.7	0.011
LVEF, %	44.3 ± 14.5	26.9 ± 6.0	<0.001
Peak VO_2_, mL/kg/min	19.5 ± 4.6	14.5 ± 4.3	0.003
**Medications**			
β-blockers	14 (93)	16 (100)	0.294
ACEI/ARB	12 (80)	16 (100)	0.060
Aldosterone antagonists	9 (60)	12 (75)	0.372
Statins	6 (40)	11 (69)	0.108
Metformin	0 (0)	1 (6)	N.A.
DPP4 inhibitors	0 (0)	3 (19)	N.A.
SGLT2 inhibitors	0 (0)	2 (13)	N.A.

Values are means ± SDs or n (%). ACEI, angiotensin-converting enzyme inhibitor; ARB, angiotensin II receptor blocker; BNP, B-type natriuretic peptide; 8-OHdG, 8-hydroxydeoxyguanosine; DPP4, dipeptidyl peptidase 4; LVEF, left ventricular ejection fraction; N.A., not applicable; NYHA, New York Heart Association; SGLT2, sodium-glucose transport protein 2; VO_2_, oxygen uptake.

### Mitochondrial respiratory capacity and ROS generation in PBMCs

**Figure 1:**
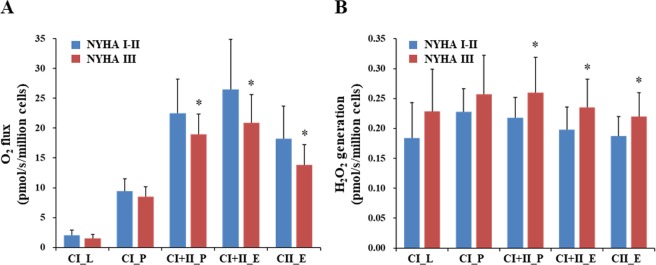
Mitochondrial respiratory capacity and mitochondrial ROS generation in PBMCs of CHF patients. **(A)** Mitochondrial respiratory capacity during each state in PBMCs in the NYHA I-II group (n = 15) and the NYHA III group (n = 16). **(B)** Mitochondrial ROS generation during each state in PBMCs in the NYHA I-II group (n = 15) and the NYHA III group (n = 16). Bar: means ± SDs. *P < 0.05. CI, complex I-linked substrates; CI + II, complex I + II-linked substrates; CII, complex II-linked substrates; E, maximal capacity of electron transfer system; H_2_O_2_, hydrogen peroxide; L, leak-state (non-ADP stimulated state); P, state 3 (ADP-stimulated state).

The NYHA III group had a lower mitochondrial respiratory capacity during state 3 respiration with complex I + II-linked substrates (CI + II_P) in PBMCs (Fig. 1A). In addition, the maximal electron transfer system (ETS) capacities with complex I + II-linked substrates (CI + II_E) and with complex II-linked substrates (CII_E) in PBMCs, respectively, were significantly reduced in the NYHA III group compared to the NYHA I-II group (Fig. 1A). Importantly, CHF patients with NYHA functional class III disease had higher mitochondrial ROS generation during state 3 respiration with complex I + II-linked substrates (CI + II_P) in their PBMCs compared to CHF patients with NYHA functional class I-II (Fig. 1B). Similarly, mitochondrial ROS generation in PBMCs was higher after addition of carbonylcyanide p-trifluoromethoxyphenylhydrazone (FCCP) with complex I + II-linked substrates (CI + II_E) and with complex II-linked substrates (CII_E) in the NYHA functional class III patients (Fig. 1B).

### Associations between mitochondrial ROS generation in PBMCs and systemic oxidative stress

**Figure 2:**
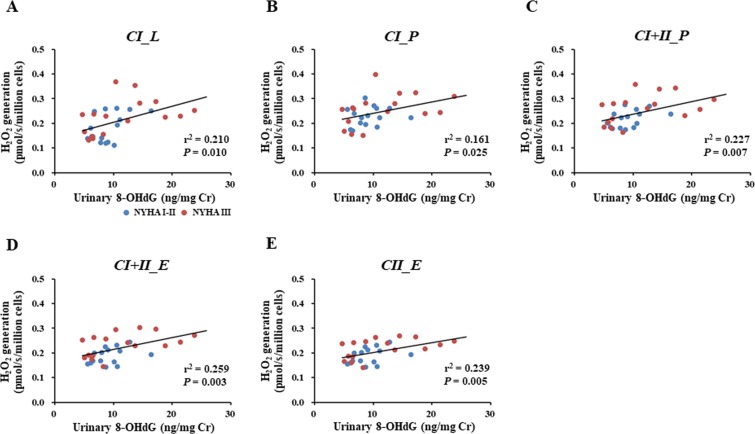
Association of systemic oxidative stress with mitochondrial ROS generation in PBMCs in CHF patients. Blue and red circles indicate CHF patients with NYHA functional class I-II (n = 15) and those with NYHA functional class III (n = 16), respectively. 8-OHdG, 8-hydroxydeoxyguanosine. Other abbreviations are as defined in the legend to Fig. 1.

Mitochondrial ROS generation in PBMCs during all respiratory states was positively correlated with urinary 8-OHdG, a biomarker of oxidative DNA damage, in CHF patients (Fig. 2), which suggested that the increased levels of mitochondrial ROS in PBMCs were associated with systemic oxidative stress in CHF patients.

### Associations between mitochondrial ROS generation in PBMCs and severity of heart failure

**Figure 3:**
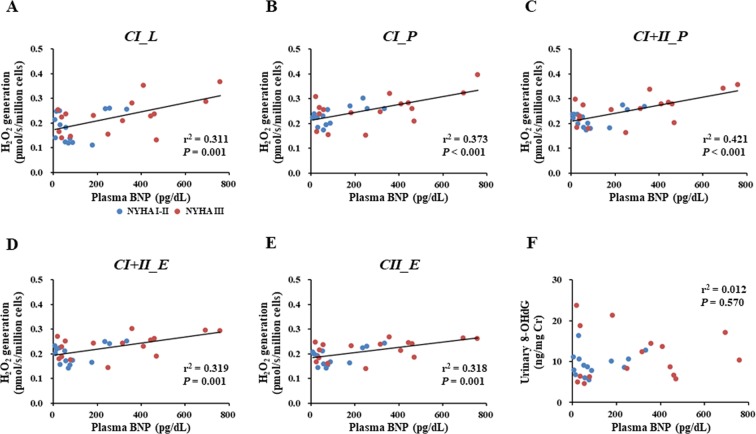
Association of plasma BNP level with mitochondrial ROS generation in PBMCs or with systemic oxidative stress in CHF patients. Associations of the plasma BNP level with mitochondrial ROS generation during each respiratory state (**A–E**) and of the plasma BNP level with systemic oxidative stress (**F**). Blue and red circles indicate CHF patients with NYHA functional class I-II (n = 15) and those with NYHA functional class III (n = 16), respectively. BNP, B-type natriuretic peptide. Other abbreviations are as defined in the legend to Fig. 1.

Mitochondrial ROS generation during all states in PBMCs was positively correlated with plasma BNP levels (Fig. 3A–E), but there was no significant correlation between urinary 8-OHdG and plasma BNP levels in CHF patients (Fig. 3F). These results indicated that PBMC mitochondrial ROS generation—but not the non-specific biomarker of systemic oxidative damage—was related to severity of HF.

### Associations between mitochondrial ROS generation in PBMCs and exercise capacity

**Figure 4:**
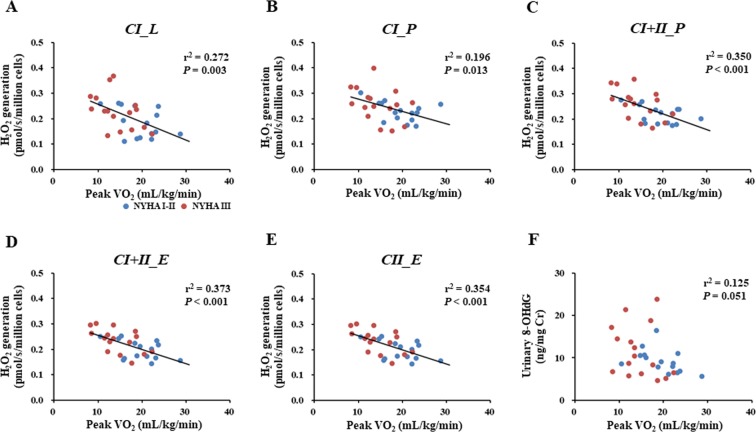
Association of exercise capacity with mitochondrial ROS generation in PBMCs or with systemic oxidative stress in CHF patients. Associations of exercise capacity with mitochondrial ROS generation during each respiratory state (**A–E**) and of exercise capacity with systemic oxidative stress (**F**). Blue and red circles indicate CHF patients with NYHA functional class I-II (n = 15) and those with NYHA functional class III (n = 16), respectively. VO_2_, oxygen uptake. Other abbreviations are as defined in the legend to Fig. 1.

ROS generation in PBMC mitochondria during all states was inversely correlated with peak VO_2_ (Fig. 4A–E), suggesting that mitochondrial ROS levels in PBMCs were associated with exercise intolerance in CHF patients. However, urinary 8-OHdG was not significantly correlated with peak VO_2_ (Fig. 4F) in CHF patients.

## Discussion

To the best of our knowledge, this is the first study to demonstrate that mitochondrial ROS generation in circulating PBMCs is markedly increased in patients with moderate-to-severe (symptomatic) CHF compared to that in patients with mild (almost asymptomatic) CHF as defined by NYHA functional class. Notably, the increased levels of mitochondrial ROS in PBMCs, but not urinary OHdG, were associated with disease severity (i.e., higher plasma BNP level) and exercise intolerance (i.e., lowered peak VO_2_) in CHF patients.

It has been shown that elevation of myocardial ROS generation causes contractile failure and structural damage, thereby leading to myocardial dysfunction in the failing heart^18–20^. In clinical settings, however, CHF is characterized by systemic illness such as endothelial dysfunction, skeletal muscle changes, and renal impairment, and systemic oxidative stress plays a crucial role in the development of CHF^21^. In this study, ROS generation in PBMC mitochondria was increased in CHF patients with NYHA functional class III compared to those with NYHA functional class I-II, and it had a weak but positive correlation with urinary 8-OHdG in CHF patients, suggesting that PBMC-derived mitochondrial ROS may be a source of systemic oxidative stress in CHF.

Unexpectedly, urinary 8-OHdG was not higher in CHF patients with NYHA functional class III compared to those with NYHA functional class I-II, which is inconsistent with a previous study^7^. However, another report found that urinary 8-OHdG levels were not higher in CHF patients of NYHA functional class III-IV taken from a non-ischemic population^22^. In our study, the percentages of non-ischemic patients in the NYHA I-II group and the NYHA III group were 73% and 56%, respectively, and these values seem to be higher than those for the general CHF population. The inclusion of a larger non-ischemic population could have influenced the results of urinary 8-OHdG in CHF patients in this study.

In this study, mitochondrial ROS generation during state 3 respiration with complex I + II-linked substrates, but not that with complex I-linked substrates, in PBMCs were increased in CHF patients with NYHA functional class III compared to those with NYHA functional class I-II. Among mitochondrial ETS complexes, complexes I and III are generally regarded as the main sources of ROS, but complex II has recently drawn much attention as an important source of ROS^23^. Indeed, the flavin site in complex II is reported to be a major contributor to mitochondrial ROS generation under physiological conditions^24^. Taken together, these findings suggest that complex II may play a role in the increased ROS generation in PBMC mitochondria in patients with advanced CHF, although we did not directly measure complex II-derived mitochondrial ROS generation in this study.

Plasma BNP is a well-established biomarker of HF that predicts clinical outcomes, including mortality and morbidity, in CHF patients^25^. We found that mitochondrial ROS generation in PBMCs, but not urinary 8-OHdG, was linked to plasma BNP levels in CHF patients, suggesting that mitochondrial ROS generation in circulating PBMCs may directly reflect the disease severity of HF rather than urinary 8-OHdG, a non-specific biomarker of oxidative damage.

CHF is a syndrome characterized by exercise intolerance with symptoms of exercise-induced dyspnea or fatigue. Indeed, exercise capacity is an independent predictor of all-cause death in CHF patients^26^. Here we showed that there was an association between increased mitochondrial ROS generation in PBMCs and lowered peak VO_2_ in CHF patients. Because determinants of VO_2_ are multifactorial, including cardiac function, pulmonary circulation, peripheral blood flow, and skeletal muscle dysfunction, a variety of abnormalities may contribute to the lowered peak VO_2_ in CHF patients. A previous study revealed that blood cell mitochondrial respiratory capacity reflects cardiac and skeletal muscle energy metabolism^27^. In the present study, we showed that increased generation of mitochondrial ROS coexisted with impaired mitochondrial respiratory capacity in PBMCs; therefore, ROS levels in PBMC mitochondria might also be linked to mitochondrial dysfunction in cardiac and/or skeletal muscles in CHF patients. Because measurements of mitochondrial ROS generation in blood cells are less invasive than those in other organs, such as cardiac muscle and skeletal muscle, ROS levels in PBMC mitochondria may be a useful biomarker not only for disease severity but also for the mitochondrial redox state in CHF.

The mechanism by which mitochondrial ROS generation in PBMCs was increased in CHF patients is not clear. One possible explanation is that an enhancement of myocardial ROS stimulates ROS generation in PBMC mitochondria via the mechanism of “ROS-induced ROS generation” upon the passage of circulating PBMCs through the heart, since it has been reported that the proportion of mitochondrial ROS-loaded blood cells is markedly higher in the coronary sinus than in the peripheral veins of CHF patients^12^. Another explanation is that systemic inflammation is involved—e.g., circulating cytokines could trigger ROS generation. Systemic inflammation is thought to be one of the primary mechanisms of CHF pathogenesis^28^. Clearly, further studies will be needed to investigate the detailed mechanism by which oxidative stress is enhanced in the PBMC mitochondria of patients with CHF.

It has previously been shown that mitochondrial respiratory capacity in PBMCs is reduced in patients with mild left ventricular hypertrophy who have a normal left ventricular systolic function with no history of worsening HF^17^. Although these patients were classified as having stage A or B HF (i.e., an early stage of HF) according to the American College of Cardiology Foundation (ACCF)/American Heart Association (AHA) guidelines^29^, they all had hypertension and/or type 2 diabetes^17^, which raises the possibility that cardiovascular risk factors affect the impaired mitochondrial respiratory capacity in PBMCs. In contrast, all of the CHF patients that we observed were classified as having either stage C or D HF according to the ACCF/AHA guidelines^29^, since they had a history of HF worsening at least once, and there was no difference in cardiovascular risk factors between the NYHA I-II and NYHA III groups. Accordingly, our findings of impaired mitochondrial respiratory capacity and increased mitochondrial ROS generation in PBMCs in CHF patients with NYHA functional class III may help elucidate the underlying mechanism of CHF progression rather than the pathogenesis of CHF, since the latter is little influenced by cardiovascular risk factors.

There are some limitations in this study. First, this is not a longitudinal study; therefore, we could not investigate whether ROS levels in PBMC mitochondria have prognostic value for CHF. Second, we could not identify the causality of the relationships among mitochondrial ROS levels of PBMCs, disease severity, and exercise intolerance in CHF patients. Third, other factors may also influence the results of PBMC mitochondrial function, because some medications such as statins and metformin alter the redox state. Fourth, we did not normalize mitochondrial ROS generation and mitochondrial respiratory capacity to mitochondrial content, and thus we cannot clearly identify whether PBMC mitochondrial dysfunction in patients with advanced CHF is attributable to intrinsic impairment of mitochondrial function or altered mitochondrial content. Finally, we did not evaluate the ROS levels of PBMC mitochondria in healthy subjects, and thus we could not examine whether patients with mild CHF already had enhanced ROS generation in their PBMC mitochondria. Since this is the first study to investigate the role of ROS in PBMC mitochondria in CHF, the present findings might be considered preliminary; they await confirmation in larger cohorts with more statistical power.

In summary, CHF patients with NYHA class III had higher levels of mitochondrial ROS in circulating PBMCs than those with NYHA class I-II, and the increased levels of mitochondrial ROS in PBMCs were associated with disease severity and exercise intolerance. Our findings provide new insights into the role of PBMC mitochondrial oxidative stress in the development of CHF.

## Methods

### Study patients

A total of 31 Japanese patients who met the Framingham Criteria and had a history of hospitalization due to HF worsening were enrolled in this study. CHF patients were recruited when they presented at the outpatient ward for a regular visit or when they were hospitalized at Hokkaido University Hospital. All the patients were stable in the compensated phase of HF. CHF patients were grouped as having either mild CHF, defined as NYHA functional class I-II (n = 15), or moderate-to-severe CHF, defined as NYHA functional class III (n = 16). Patients with chronic inflammatory disease, cancer, or chronic kidney disease with current hemodialysis were excluded. Written informed consent was obtained from each patient before participation in the study. This study was approved by the ethical committee of Hokkaido University Hospital and was registered in the UMIN Clinical Trials Registry: UMIN000022564. All investigations conformed to the principles outlined in the Declaration of Helsinki.

### Study protocol

Peripheral venous blood and urine samples were collected from all subjects after a 10-h overnight fast. To isolate PBMCs, a Ficoll-Paque gradient medium (GE Healthcare Life Sciences, Piscataway, NJ) was used according to the manufacturer’s protocol for analysis of mitochondrial respiratory capacity and mitochondrial ROS generation in PBMCs on the same day. Portions of each obtained blood sample and urine sample were stored at −80 °C for later analysis of systemic oxidative stress. Plasma levels of BNP were determined by routine in-house analyses. Cardiac function of CHF patients was evaluated by echocardiography. The LVEF was measured from apical 4- and 2-chamber images using the biplane method of disks. To assess peak VO_2_, CHF patients underwent symptom-limited cardiopulmonary exercise testing using an upright bicycle ergometer with a ramp protocol, and respiratory gas analysis was simultaneously performed with a breath-by-breath apparatus (Aeromonitor AE-310S; Minato Medical Science, Osaka, Japan).

### Mitochondrial respiratory capacity

The mitochondrial respiratory capacity was measured with a high-resolution respirometer (Oxygraph-2k; Oroboros Instruments, Innsbruck, Austria) at 37 °C within 6 h after blood collection, according to a previously described method with minor modifications^17^.

Isolated PBMCs were suspended in mitochondrial respiration medium (MiR05) containing 0.5 mM ethylene glycol tetraacetic acid (Sigma-Aldrich, St. Louis, MO), 3 mM MgCl_2_ (Sigma-Aldrich), 20 mM taurine (Sigma-Aldrich), 10 mM KH_2_PO_4_ (Merck Millipore, Darmstadt, Germany), 20 mM HEPES (Sigma-Aldrich), 110 mM D-sucrose (Carl-Roth, Karlsruhe, Germany), 60 mM K-lactobionic acid (Sigma-Aldrich), and 1 g/L essentially fatty acid-free bovine serum albumin (Sigma-Aldrich), and were prepared to a final cell density of 2 × 10^6^ cells per milliliter.

After the addition of PBMC suspension to the chamber of the respirometer, digitonin (2 μmol/L) was added to permeabilize the PBMCs, and the multiple substrate-uncoupler-inhibitor-titration (SUIT) protocol was applied to evaluate the mitochondrial respiratory capacity during each state. The respiration of CI_L (i.e., respiration during the leak state or state 2 with complex I-linked substrates) was measured after adding malate (final concentration, 2 mmol/L), pyruvate (5 mmol/L) and glutamate (10 mmol/L). Then, adenosine diphosphate (ADP; 5 mmol/L) was added to measure the respiration of CI_P (i.e., ADP-stimulated respiration or respiration during state 3 with complex I-linked substrates). Succinate (a complex II-linked substrate) was added with a titration protocol (10 mmol/L increment) until reaching maximal respiration to measure CI + II_P (i.e., ADP-stimulated respiration or respiration during state 3 with complex I + II-linked substrates). CI + II_E (maximal capacity of ETS with complex I + II-linked substrates) was evaluated after titration of FCCP (an uncoupler, 0.5 µmol/L increments), and CII_E (maximal capacity of ETS with complex II-linked substrates) was evaluated after inhibition of complex I with rotenone (0.5 μmol/L).

The respiratory rate (i.e., the O_2_ consumption rate or O_2_ flux) values are expressed as pmol/s/million cells of PBMCs. DatLab software (Oroboros Instruments) was used for data acquisition and data analysis.

### Mitochondrial ROS generation

We measured mitochondrial ROS generation simultaneously with the mitochondrial respiratory capacity in the permeabilized PBMCs using a spectrofluorometer (Fluorescence LED2-Module; Oroboros Instruments) equipped with a respirometer, as previously described^30^. Mitochondrial ROS were evaluated after the conversion of mitochondrial superoxide (O_2_^−^) into hydrogen peroxide (H_2_O_2_) by the addition of superoxide dismutase (SOD). Before the permeabilization of PBMCs, we added SOD (5 U/mL), horseradish peroxidase (1 U/mL), and Amplex® UltraRed reagent (10 µmol/L; Thermo Fisher Scientific, Waltham, MA) to the chamber of the respirometer. H_2_O_2_ reacts with Amplex UltraRed in a 1:1 stoichiometry catalyzed by horseradish peroxidase, which yields the fluorescent compound resorufin. The excitation wavelength was 525 nm, and fluorescence detection was at 587 nm. The fluorescence of resorufin was continuously monitored during the SUIT protocols (along with the measurements of mitochondrial respiratory capacity).

The H_2_O_2_ generation rate was calibrated by the titration of H_2_O_2_ in 0.1 µmol/L increments before and after each substrate addition, in order to eliminate the possible interference of substrates. The H_2_O_2_ generation rate is expressed as pmol/s/million cells of PBMCs.

### Systemic oxidative stress

To assess systemic oxidative stress, we measured urinary 8-OHdG, a biomarker of oxidative DNA damage, with an enzyme-linked immunosorbent assay kit (Highly sensitive 8-OHdG check; Japan Institute for the Control of Aging, Shizuoka, Japan). The values were normalized to the urinary level of creatine.

### Statistical analysis

Data are expressed as means ± standard deviations (SDs). We used Student’s *t-*tests for continuous variables and chi-square tests for categorical variables to compare the data between two groups. We conducted a Pearson’s correlation analysis to determine linear relationships between continuous variables. Statistical analyses were performed using GraphPad Prism 7.0a software (GraphPad Software, San Diego, CA), and significance was defined as *P* < 0.05.
